# Distinct partitioning of ALS associated TDP-43, FUS and SOD1 mutants into cellular inclusions

**DOI:** 10.1038/srep13416

**Published:** 2015-08-21

**Authors:** Natalie E. Farrawell, Isabella A. Lambert-Smith, Sadaf T. Warraich, Ian P. Blair, Darren N. Saunders, Danny M. Hatters, Justin J. Yerbury

**Affiliations:** 1 Illawarra Health and Medical Research Institute, Wollongong, NSW 2522 Australia; 2 Faculty of Science, Medicine and Health, University of Wollongong, Wollongong, NSW 2522 Australia; 3 The University of Melbourne, Melbourne, VIC 3010, Australia; 4 Australian School of Advanced Medicine, Macquarie University, Sydney, NSW 2109, Australia; 5 Cancer Division, Garvan Institute of Medical Research, The Kinghorn Cancer Centre, Darlinghurst NSW 2010, Australia; 6 St Vincent’s Clinical School, UNSW Medicine.

## Abstract

Amyotrophic lateral sclerosis is a rapidly progressing neurodegenerative disease associated with protein misfolding and aggregation. Most cases are characterized by TDP-43 positive inclusions, while a minority of familial ALS cases are instead FUS and SOD1 positive respectively. Cells can generate inclusions of variable type including previously characterized aggresomes, IPOD or JUNQ structures depending on the misfolded protein. SOD1 invariably forms JUNQ inclusions but it remains unclear whether other ALS protein aggregates arise as one of these previously described inclusion types or form unique structures. Here we show that FUS variably partitioned to IPOD, JUNQ or alternate structures, contain a mobile fraction, were not microtubule dependent and initially did not contain ubiquitin. TDP-43 inclusions formed in a microtubule independent manner, did not contain a mobile fraction but variably colocalized to JUNQ inclusions and another alternate structure. We conclude that the RNA binding proteins TDP-43 and FUS do not consistently fit the currently characterised inclusion models suggesting that cells have a larger repertoire for generating inclusions than currently thought, and imply that toxicity in ALS does not stem from a particular aggregation process or aggregate structure.

Amyotrophic Lateral Sclerosis (ALS) is characterised by the progressive and selective death of upper and lower motor neurones in the motor cortex and spinal cord. This leads to loss of muscle control, muscle atrophy and invariably death, generally within 3–5 years of diagnosis. The cause(s) of most cases of ALS (sporadic ALS; sALS) remain undefined, however, approximately 5–10% of cases are inherited (familial ALS; fALS). Mutations in genes now known to cause ALS include SOD1^1^, FUS/TLS^2^,^3^, VAPB^4^, TARDBP^5^,^6^, OPTN^7^, VCP^8^, SQSTM1^9^, UBQLN2^10^, PFN1^11^, MATR3^12^ and hexanucleotide expansions in C9ORF72^13^.

In common with other neurodegenerative diseases such as Alzheimer’s disease, Creutzfeldt-Jakob disease, Parkinson’s disease, Huntington’s disease^14^, there is growing evidence that a correlation exists between protein aggregate formation and neuronal loss in ALS spinal cord^15^,^16^,^17^,^18^,^19^. Indeed, the progression of disease has been proposed to be due to propagation of protein misfolding and aggregation^20^. Inclusions associated with neurodegenerative disease are made up of insoluble proteinaceous material that consist mainly of one main constituent^14^, but also are composed of a variety of proteins, including molecular chaperones^21^,^22^ and the proteasome^23^. It is unclear how these additional components become part of the inclusions, but at least some may be there due to the functional interaction, such as chaperone and degradation machinery, with misfolded proteins in the aggregates^24^. In Huntington’s disease, huntingtin with expanded poly glutamine repeats (polyQ) predominates in neuronal inclusions. In Parkinson’s disease, inclusions known as Lewy bodies are thought to invariably consist mainly of α-synuclein. Ubiquitin positive round and skein-like inclusions in most cases of ALS are immunoreactive for TDP-43^25^,^26^, the exceptions are SOD1 and FUS familial cases of ALS which are associated with SOD1 and FUS positive inclusions respectively^2^,^3^,^27^.

More generally, previous work has identified common structures created during aggregation of a range of proteins, such as soluble oligomeric aggregates, that can be toxic when applied to cells^28^. However, in ALS pathology it remains unclear if the inclusions, or other aggregate form, are themselves toxic or if they are a symptom of proteotoxicity, reflecting a cellular response/adaptation to misfolded protein. Within the cellular milieu, protein aggregates or inclusions can be generated by several distinct pathways. Originally, cell driven, microtubule dependant juxtanuclear inclusions of CFTR ensheathed by vimentin were discovered and termed aggresomes^29^. These may be related to the **ju**xta**n**uclear **q**uality control compartment (referred to as JUNQ^30^) later observed in yeast and mammalian cells. JUNQ-like inclusions are enriched in ubiquitylated proteins, proteasome subunits, chaperones such as Hsp70 and have a mobile fraction^30^,^31^. In addition, an **i**nsoluble **p**rotein **d**eposit (IPOD) compartment has been observed in yeast and mammalian cells and is a dense, immobile compartment consisting of insoluble protein aggregates that (at least initially) are not ubiquitinated^30^,^32^. While postmortem studies have identified ALS inclusions that are either TDP-43, FUS or SOD1 immunoreactive, a thorough characterisation and comparison of how these inclusions arise has not been performed. While misfolded mutant SOD1 is consistently associated with JUNQ-like inclusions as reported previously^30^,^33^, we now report for the first time that while FUS and TDP-43 can form iPOD and/or JUNQ-like inclusions respectively they can also partition to inclusions that are distinct from SOD1 inclusions, distinct from each other, and from other described inclusions types. These data may suggest that cells have a broader array of pathways for generating proteinacious inclusions than originally thought, and imply that toxicity in ALS does not stem from a particular aggregation process or aggregate structure.

## Results

### Distinct partitioning of ALS associated mutant SOD1 from TDP-43^wt^ aggregates

**Figure 1:**
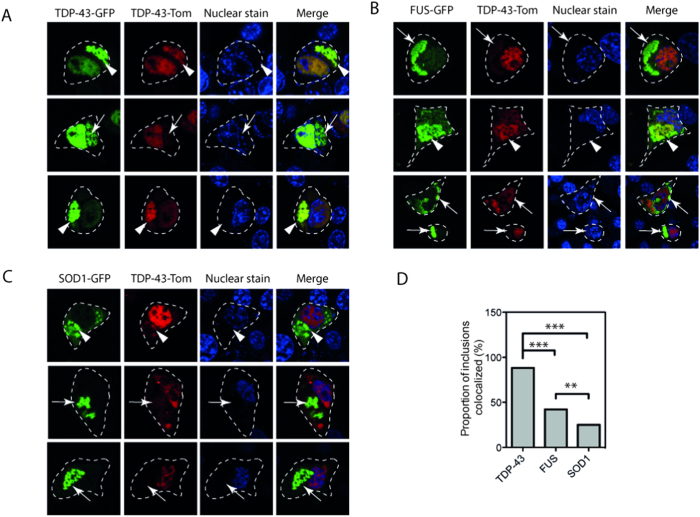
Aggregated mutant TDP-43 and FUS recruit TDP-43^wt^. NSC-34 cells were transiently transfected with TDP-43^wt^-tomato and examined by confocal microscopy after 48 hours. (**A**) Mutant TDP-43^M337V^-GFP coexpressed with TDP-43^wt^-tomato. (**B**) Mutant FUS^R495X^ co-expressed with TDP-43^wt^-tomato. (**C**) SOD1^A4V^ co-transfected with TDP-43^wt^ tomato. White arrow heads indicate areas of colocalization of GFP and tomato tagged protein. White arrows indicate distinct inclusion structures containing one tagged protein only. (**D**) Proportion of cells with aggregates where both mutant TDP-43, FUS and SOD1 colocalize with TDP-43^wt^. Data is combined from 3 independent experiments, Z-tests were used to compare differences in proportions **p < 0.01, ***p < 0.001.

Given that many ALS inclusions contain TDP-43^wt^, we sought to examine whether overexpression of mutant TDP-43, FUS or SOD1 would induce TDP-43 aggregation and if so whether the aggregates accumulate in distinct cellular compartments. We used TDP-43^wt^-tomato^34^, which under basal conditions remains predominantly in the nucleus, as a reporter for TDP-43 recruitment into aggregates. Aggregated TDP-43 has previously been shown to recruit TDP-43^wt^ into cytoplasmic inclusions^35^. Consistent with this, we observe that co-expression of TDP-43^M337V^-GFP and TDP-43^wt^-tomato produces cytosolic inclusions (defined as fluorescent foci > 2 μm) positive for both mutant and TDP-43^wt^ in approximately 88% of cells expressing both constructs that contain inclusions (Fig. 1A,D; white arrow heads). However, while the bulk of TDP-43^M337V^ is retained in the cytosol in inclusions, co-localization of TDP-43^wt^ with these inclusions did not always deplete the nucleus of TDP-43^wt^. Indeed, in some instances inclusion formation of TDP-43^M337V^ did not recruit TDP-43^wt^ (Fig.1A, white arrows).

Although FUS can be found associated with ubiquitin positive inclusions in sALS^36^, FUS positive inclusions in mutant FUS fALS cases are not TDP-43 positive^37^. In addition, it has been suggested that FUS and TDP-43 may have different aggregation pathways in cells^38^. In the current study, expression of FUS^R495X^ -GFP lead to inclusions that when co-expressed with TDP-43^wt^-tomato resulted in co-aggregation of mutant FUS and TDP-43^wt^ (Fig. 1B,C, white arrow heads; ~ 42% of cells containing inclusions of FUS^R495X^ and TDP-43^wt^). This suggests that at least in some cases, and under the conditions used here, FUS recruits TDP-43 to the same type of aggregates in neuron-like cells. Similar to expression of mutant TDP-43, not all FUS^R495X^ inclusions recruited TDP-43^wt^ (Fig. 1B, white arrows).

In sporadic ALS the co-localization of SOD1 with TDP-43 positive inclusions is rare, and SOD1 fALS cases are characterized by inclusions positive for SOD1 but negative for TDP-43^39^. Consistent with these data, our cell model of overexpression of SOD1^A4V^ rarely resulted in the aggregation of both SOD1^A4V^ and TDP-43^wt^ (Fig. 1C; 1 occurrence in every 140 cells containing SOD1). However, in the event that TDP-43 aggregation occurred in the presence of SOD1^A4V^ aggregates ~ 25% of these inclusions colocalized. Notably, there was a significant difference between the proportion of cells containing colocalization of TDP-43^wt^ in cells containing aggregates of TDP-43^M337V^, FUS^R495X^, SOD1^A4V^ and TDP-43^wt^ (z-test; n = 73, 165, 79 respectively; p < 0.01) suggesting different mechanisms of aggregation in each case.

Given the predominant co-aggregation of mutant TDP-43 and FUS but not SOD1 with TDP-43^wt^, we examined the potential role of RNA in the construction of these inclusions. Firstly, we used actinomycin D to supress RNA synthesis (Fig. S1C) to determine if reducing overall RNA levels would alter inclusion formation. Treatment of cells with actinomycin D overnight did not significantly change the number of inclusions formed in cells expressing TDP-43^M337V^, FUS^R495X^ or SOD1^A4V^ (Fig. S1). Next we lysed cells containing inclusions and then treated lysates with RNase. We reasoned that if TDP-43 and FUS were bound to inclusions at least partly via interactions with RNA then treatment would disrupt inclusions. In the absence of RNase inclusions were trapped in our filter trap assay (Fig. S1D,E). Similar levels of TDP-43^M337V^, FUS^R495X^, and SOD1^A4V^ were found trapped regardless of RNase treatment (Fig. S1D,E), suggesting inclusion formation was independent of RNA.

### Microtubule disruption affects SOD1 inclusion formation but not TDP-43 and FUS

**Figure 2:**
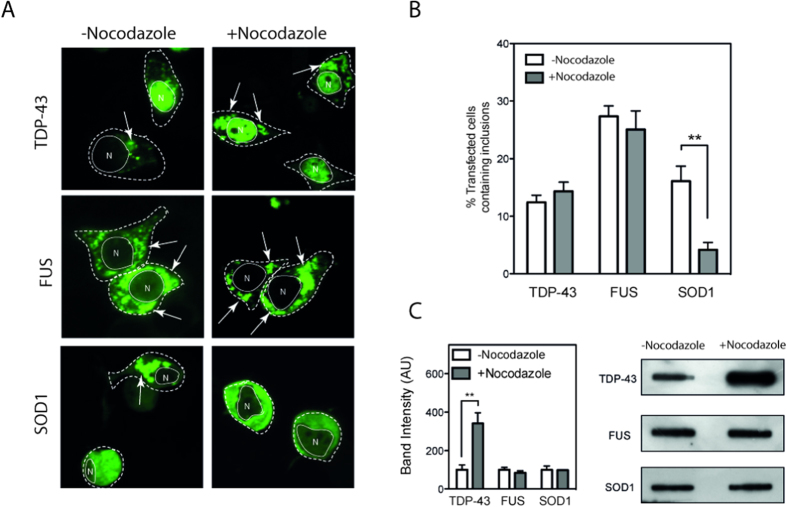
Microtubule destabilization prevents SOD1 inclusions but not TDP-43 and FUS inclusions. NSC-34 cells were transiently transfected with mutant TDP-43, FUS, or SOD1-GFP and after 24 hours incubated with or without 33 μM nocodazole. (**A**) Confocal images of cells expressing GFP fusion proteins in the presence or absence of nocodazole. Dotted white line represents cell outline, solid white line represents nuclear outline obtained from transmission image (denoted N), white arrows indicate inclusions. (**B**) Quantification of the proportion of cells with inclusions in transfected cells. At least 6 fields of view from each timepoint were counted (minimum 30 cells per field) and scored. Experiments were performed 3 times and bar charts represent mean and standard deviation. ** indicates p < 0.01 (**C**) After treatment with nocodazole cells were lysed and lysates were used in filter trap assays. Trapped material represents aggregates. Western blotting of resulting filter trap assay and quantification using imageJ. Experiments were performed 3 times and bar charts represent mean and standard deviation. **indicates p < 0.01.

Previous work indicates that microtubule disruption promotes TDP-43 inclusions^40^ and halts the formation of a very specific FUS inclusion type formed by the 1–359 FUS fragment^41^. Similarly, aggresomes, IPOD and JUNQ structures are suppressed when microtubules are disrupted^29^,^30^. To compare the effects of destabilization of microtubules on ALS inclusions we treated NSC-34 cells transfected with mutant FUS, TDP-43 or SOD1 fused to GFP with microtubule destabilizer nocodazole. We observed large cytoplasmic inclusions that are cloud or sponge-like in appearance (referred to as conglomerate in ALS pathology) in approximately 15% of SOD1^A4V^ -GFP transfected cells (Fig. 2A,B) but after nocodazole treatment this was significantly reduced (p < 0.01; Student’s t-test) to fewer than 5% of transfected cells had large inclusions. The remaining cells did not appear to have smaller granular foci as observed in other aggregating systems such as occurs in the Htt^42^ model but remained in a diffuse pattern of fluorescence. This is consistent with previous observations of mutant SOD1 forming JUNQ-like aggresomes that are microtubule dependent^43^. In contrast, NSC-34 cells transfected with either TDP-43^M337V^ or FUS^R495X^ showed no significant difference in percentage of inclusions present regardless of nocodazole treatment (Fig. 2A,B). This suggests that neither FUS nor TDP-43 aggregate in a manner consistent with aggresomes, IPOD or JUNQ structures. However, it must be noted that although there is evidence that IPOD–like inclusions require intact microtubules to aggregate, evidence also exists to show that polyQ-104 protein aggregates similarly in the presence or absence of nocodazole^44^. To complement image analysis we also performed filter trap assays to examine any difference in total trappable aggregated protein under the same conditions. We found that although the number of SOD1 inclusions decreased upon nocodazole treatment (Fig. 2B, p < 0.05; Student’s t-test), total aggregation measured by filter trap did not (Fig. 2C). Similarly, FUS aggregation did not change after nocodazole treatment. In contrast, total trappable TDP-43 aggregates significantly increased upon nocodazole treatment (Fig. 2C; p < 0.01; Student’s t-test).

### TDP-43 aggregates are immobile

**Figure 3:**
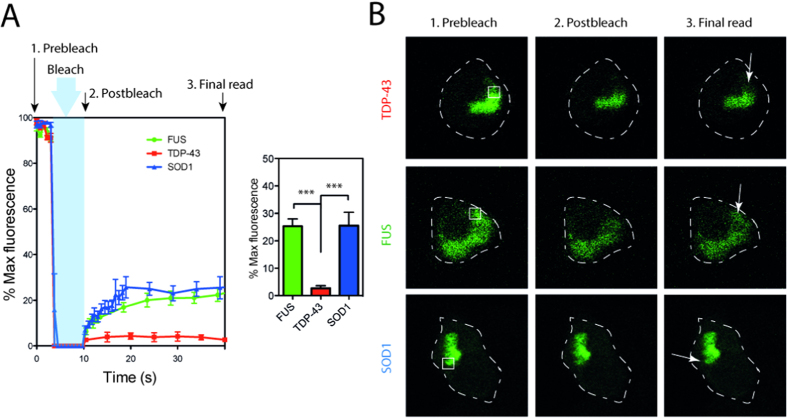
SOD1 and FUS inclusions contain a mobile fraction. FRAP analysis of inclusions formed in NSC-34 cells expressing mutant TDP-43, FUS or SOD1-GFP. (**A**) Mean fluorescence intensity (from within the ROI) plotted over time. Prebleach intensity was recorded, and recovery was recorded for up to 40 s. Results are means and standard deviation from n = 10. Mean fluorescence at 40 s is quantified in histogram. ***indicates p < 0.001. (**B**) Representative confocal images of prebleach, post bleach and recovery endpoint. ROI is marked in white, timing of image relative to data collection is marked in panel A.

IPOD structures are thought to be predominantly immobile, while JUNQ and aggresome structures contain a mobile fraction^31^,^45^. Here we used fluorescence recovery after photo bleaching (FRAP) to examine the mobility of molecules within ALS inclusions. Using a high powered laser pulse we bleached a region of GFP positive inclusions that corresponded to < 25% of any given inclusion and then recorded the subsequent fluorescence over 40 s. We found that there was minimal fluorescence recovery after bleaching TDP-43^M337V^ inclusions (Fig. 3A,B) confirming recent observations^46^. In contrast, our data suggests that both SOD1 and FUS inclusions recover approximately 25% of their original fluorescence, significantly more recovery than TDP-43 (Tukey’s multiple comparison test; p < 0.001), suggesting a mobile fraction or porous structure within the inclusions (Fig. 3A,B). The recovery patterns of each of the aggregates was similar to that of the soluble protein tested in both wt and mutant expressing cells (Fig. S2). Therefore we cannot rule out the possibility that the protein species diffusing in to the inclusion are soluble species from outside of the aggregate and do not represent diffusion from within the aggregate itself.

### FUS but not TDP-43 and SOD1 co-aggregate with IPOD substrate Huntingtin_ex1_46Q

**Figure 4:**
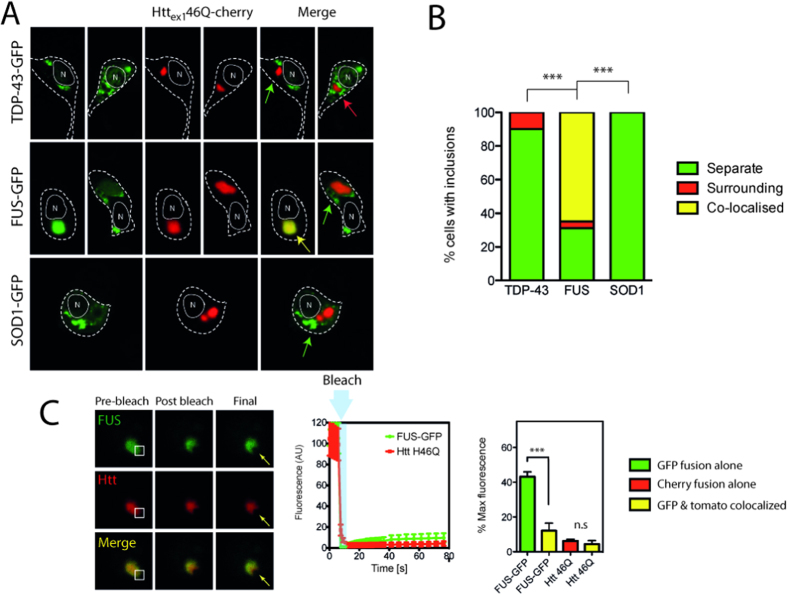
SOD1 and TDP-43 inclusions are distinct from iPOD. Cells were transfected with Htt_ex1_46Q-mcherry and either mutant TDP-43, FUS or SOD1-GFP. The cells were imaged 48 h after transfection (**A**) and then the number of inclusions colocalized or surrounding the Htt iPOD quantified (**B**). Green arrows indicate distinct and separate inclusions, red arrows indicate GFP tagged inclusions surrounding Htt inclusions, yellow arrows indicate colocalization. A minimum of 100 cells with inclusions were counted in each case and the experiment was repeated n = 2. A z-test of proportions was conducted on combined dataset and ***denotes p < 0.001. (**C**) FRAP measurements were performed on Htt-FUS positive inclusions. White box indicates ROI, arrow indicates bleached area. Mean fluorescence intensity was followed for 80 s and data shown are means and standard deviations n = 6. Histogram is data from FUS and Htt colocalized at 80 s compared to that of either FUS-GFP or Htt-RFP alone. ***indicates p < 0.001.

Proteins that aggregate in to insoluble deposits can be recruited in to the same IPOD structure in cells regardless of their amino acid sequence^31^,^33^. To test whether ALS mutants would form IPOD-like structures we co-transfected NSC-34 cells with IPOD substrate Huntingtin_ex1_46Q fused to cherry (Htt_ex1_46Q-cherry) and either TDP-43^M337V^, FUS^R495X^, or SOD1^A4V^ GFP fusions. Expression of Htt_ex1_46Q consistently generated a round IPOD-like inclusion in the cytoplasm or nuclei in NSC-34 cells (Fig. 4A). Consistent with previous work, co-expression of Htt_ex1_46Q-cherry with mutant SOD1 produced inclusions of SOD1 distinct from Htt_ex1_46Q-cherry inclusions in 100% of cells expressing both proteins (Fig. 4A,B)^30^,^31^,^33^. When TDP-43^M337V^ was co-expressed with Htt_ex1_46Q-cherry, inclusions were predominantly separate with no co-localization (Fig. 4A green arrow). However, in ~ 10% of cells containing aggregates of both TDP-43 and Htt the TDP-43^M337V^ aggregates were tightly packed around the dense Htt core (Fig. 4A red arrow). In contrast, a statistically significant increase in FUS aggregates, compared to TDP-43 and SOD1, (n = 46; compared to SOD1 (n = 42) and TDP-43 (n = 45); z-test, p < 0.001) co-localized to Htt_ex1_46Q-cherry IPOD-like structures (Fig. 4A yellow arrow). The remaining FUS aggregates were found to be similar to those observed for TDP-43 with separate inclusions, with a small percentage of those tightly packed around the Htt_ex1_46Q-cherry core (Fig. 4B).

Given that we had found that FUS aggregates contained a mobile fraction, and that previous work had demonstrated that IPOD structures contain immobile aggregates, we used FRAP to investigate if FUS aggregates remained mobile in IPOD structures. When a region of an inclusion that contained both FUS and Htt_ex1_46Q-cherry was bleached we found that neither FUS nor Htt_ex1_46Q-cherry fluorescence recovered significantly (Fig. 4C), indicating that FUS was mostly immobile in IPOD structures. Under the same conditions, FUS-only aggregates recovered around 40% of the bleached fluorescence (Fig. S3C) and Htt_ex1_46Q-cherry was immobile with no fluorescence recovery (Fig. S3A). These results indicate a significant decrease in recovery of FUS fluorescence in the presence of Htt aggregates (Fig. 4C; p < 0.001; Student’s t-test). In the cases where colocalization of Htt and FUS occurred, we observed a pattern of fluorescence in which ~ 80% of total FUS fluorescence and ~ 90% of total Htt was consistently associated with the dual labelled inclusions (Fig. S4A).

### TDP-43 and FUS variably co-aggregate with SOD1^A4V^ JUNQ

**Figure 5:**
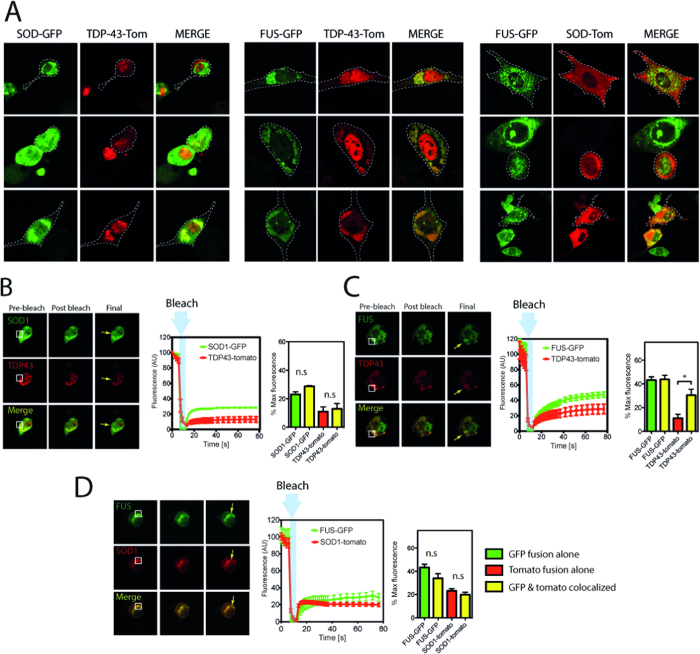
Mutant TDP-43 can co-aggregate to JUNQ like SOD1 positive structures. NSC-34 cells were cotransfected with combinations of mutant SOD1, TDP-43 and FUS and imaged after 48 h (**A**). Mutant TDP-43 could be found colocalized with SOD1 in some cases. (**B**) Mutant SOD1 and TDP-43 positive inclusions were analyzed using FRAP. White box indicates ROI, arrow indicates bleached area. Mean fluorescence intensity was followed for 80 s and data shown are means and standard deviations n = 6. Histogram is data from SOD1 and TDP-43 colocalized at 80 s compared to that of either SOD1-GFP or TDP-43-tomato alone. (**C**) Mutant FUS and TDP-43 positive inclusions were analyzed using FRAP. White box indicates ROI, arrow indicates bleached area. Mean fluorescence intensity was followed for 80 s and data shown are means and standard deviations n = 6. Histogram is data from FUS and TDP-43 colocalized at 80 s compared to that of either FUS-GFP or TDP-43-tomato alone. *indicates p < 0.05. (**D**) Mutant FUS and SOD1 positive inclusions were analyzed using FRAP. White box indicates ROI, arrow indicates bleached area. Mean fluorescence intensity was followed for 80 s and data shown are means and standard deviations n = 9. Histogram is data from FUS and SOD1 colocalized at 80 s compared to that of either FUS-GFP or SOD1-tomato alone.

SOD1 is a well-characterized JUNQ substrate^30^,^31^,^33^. To test whether TDP-43 or FUS might be JUNQ substrates we co-transfected TDP-43^M337V^ or FUS^R495X^ with SOD1^A4V^ fusion proteins. In cells that contained inclusions of both TDP-43 and SOD1 we observed that inclusions were distinct 50% of the time, the remaining cells contained inclusions that were positive for both TDP-43 and SOD1 (Fig. 5A). In the cases where colocalization occurred, we observed a pattern of fluorescence in which ~ 80% of total SOD1 fluorescence was consistently associated with the dual labelled inclusions (Fig. S4B). In contrast, the amount of TDP-43 present in these inclusions was significantly lower in comparison to SOD1 (p < 0.001; Student’s t-test) and ranged from ~ 50% to ~ 70% (Fig. S4B). This suggested to us that under certain specific conditions TDP-43 could be a JUNQ substrate. Similarly, while FUS did not form round dot like inclusions that were observed when co-expressed with Htt_ex1_46Q-cherry, there was co-localization with SOD1^A4V^ inclusions in approximately 30% of cells containing inclusions of both SOD1 and FUS (Fig. 5A). In the cases where there was inclusion colocalization ~ 80% of the total cellular SOD1 and FUS was present in the inclusion suggesting an active co-aggregation process (Fig. S4C). When TDP-43^M337V^ and FUS^R495X^ were aggregated in the same cell around 85% of the inclusions were positive for both TDP-43 and FUS (Fig. 5A). In a manner similar to that of SOD1 we observed that colocalized inclusions consistently contained ~ 70% of total cellular FUS, these same inclusions variably contained a significantly lower amount (p < 0.05, Student’s t-test), ~ 50% of the TDP-43 signal (Fig. S4D), consistent with TDP-43 aggregation being a secondary process.

Since we had observed TDP-43 inclusions to be immobile and a significant proportion of TDP-43 inclusions co-localized with SOD1 inclusions we performed FRAP to examine the mobility of these proteins in dual labeled inclusions. When bleached with 488 nm laser, TDP-43^M337V^ remained immobile while SOD1^A4V^-GFP signal partially diffused back in to the bleached area—consistent with a previously observed mobile fraction (Fig. 5B). In contrast, FRAP measurements on inclusions that contained both FUS^R495X^ and TDP-43^M337V^ indicated that when TDP-43 was recruited in to FUS positive inclusions a proportion of the TDP-43 signal was recovered, consistent with a mobile fraction (Fig. 5C). This is consistent with TDP-43 being a secondary aggregator in these inclusions. When inclusions contained both FUS^R495X^ and SOD1^A4V^ both FUS and SOD1 retained a diffusible fraction (Fig. 5D).

**Figure 6:**
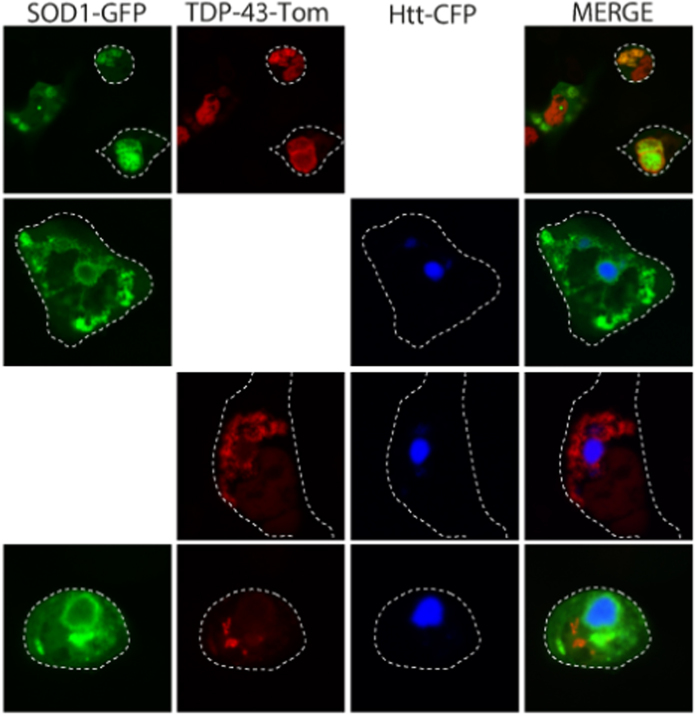
Triple transfection reveals 3 distinct inclusions. NSC-34 cells were cotransfected with combinations of SOD1, TDP-43 and Htt including a triple transfection of all three plasmids and imaged after 48 h.

Given the different structural properties of TDP-43 and SOD1 inclusions, but their partial overlap when co-expressed, we performed triple transfections of TDP-43^M337V^-tomato, SOD1^A4V^ -GFP and Htt_ex1_46Q-Cerulean fluorescent protein. In this scenario, TDP-43 co-localized with SOD1^A4V^ aggregates in some instances, but never co-localized with Htt_ex1_46Q-Cerulean inclusions (Fig. 6). In some cases the cells were able to compartmentalize all three inclusions from one another. In these cases many of the TDP-43 inclusions appeared to be in the nucleus or very close to the nuclear envelope (Supplementary Material).

### FUS and TDP-43 inclusions are ubiquitylated late compared to SOD1 inclusions

**Figure 7:**
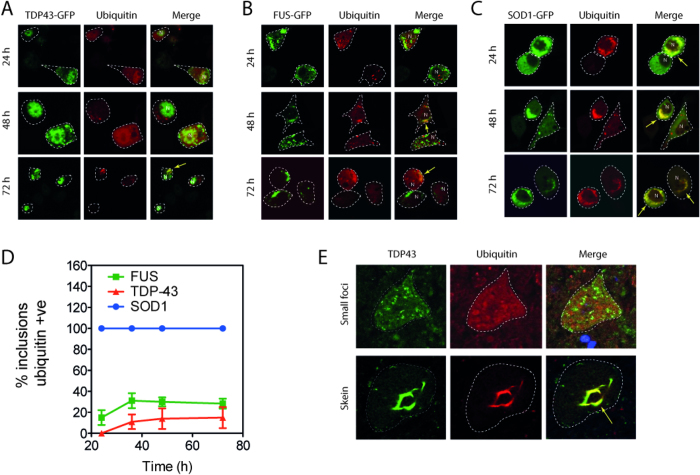
Ubiquitin associates with TDP-43 and FUS inclusions late compared to SOD1. NSC-34 cells were transiently transfected with mutant TDP-43, FUS, or SOD1-GFP with mRFP-ubiquitin and examined by confocal microscopy at various times after transfection (**A**–**C**). Quantification of proportion of inclusions in dual transfected cells that contain both GFP fusion proteins and ubiquitin (**D**). 8 fields of view from each timepoint were counted (minimum 30 cells per field) and scored. Experiment was performed in triplicate and is representative of 2 independent experiments. (**E**) Human ALS post mortem tissue was stained for both TDP-43 and ubiquitin. 40 inclusions were imaged across two cases of sporadic ALS. Representative images of small foci (not colocalizing with ubiquitin) and large skeins that colocalize to ubiquitin staining are shown.

Ubiquitin positive inclusions are a hallmark of ALS, however the mechanism and dynamics of ubiquitylation and inclusion formation cannot be determined from postmortem tissue. Recent work on Huntingtin systems shows that although ubiquitylated Htt positive inclusions are present in post mortem tissue, in cell culture systems ubiquitin co-localizes to inclusions relatively late^32^. To compare the association of ubiquitin with ALS inclusions we initially co-transfected NSC-34 cells with mRFP-ubiquitin and SOD1^A4V^, TDP-43^M337V^ or FUS^R495X^ GFP fusions. SOD1^A4V^ inclusions were always observed to co-localize with ubiquitin starting at our earliest observations 24 hours after transfection (Fig. 7C,D). In contrast, TDP-43^M337V^ and FUS^R495X^ did not always co-localize with ubiquitin with around 15 and 30% of inclusions respectively becoming positive for mRFP-ubiquitin after 72 h (Fig. 7A,B,D). To confirm this was not an artefact of the co-expression with mRFP-ubiquitin fusion we performed ubiquitin- immunostaining on cells expressing mutant FUS, TDP-43 and SOD1-GFP alone and observed similar ubiquitin co-localization patterns at 48–72 hours (Fig. S5).

Our results in cell culture are seemingly incongruous with those from post mortem ALS spinal cord sections that suggest that ubiquitin is a near universal marker of TDP-43 inclusions. To examine the relationship between TDP-43 and ubiquitin in human ALS we stained human ALS post mortem spinal cord sections and observed that 38 out of 40 TDP-43 inclusions (from 2 SALS cases) co-localized with ubiquitin. Strikingly, the 2 out of 40 inclusions that did not co-localize with ubiquitin (Fig. 7E) were smaller and more punctate (similar to the inclusions seen in our cell models after 48 h), while the ubiquitin-positive inclusions were generally extremely large skein-like inclusions (Fig. 7E). Interestingly, we rarely observed skein-like inclusions in our cell culture models, suggesting that skeins may develop over a long period of time.

Given the late redistribution of ubiquitin we tested whether the ubiquitin negative inclusions might co-localize to autophagy marker LC3. We observed that while both TDP-43 and FUS inclusions were not co-localized with LC3 they were found adjacent to LC3 positive foci. In contrast, there was no observable relationship between SOD1 inclusions and LC3 (Fig. S6).

## Discussion

The role of protein aggregates in the pathology of a range of neurodegenerative diseases such as Alzheimer’s disease, Parkinson’s disease, and Huntington’s disease has gained increasing support^14^,^47^. Generally in these cases there is one dominant pathogenic protein or peptide, such as Aβ, tau, α-synuclein, and huntingtin that misfolds and aggregates potentially causing cellular damage along the way. ALS is also associated with protein misfolding and aggregation, however although all cases are associated with ubiquitin-positive inclusions, the make up of these inclusions varies depending on their location in the CNS and the underlying genetic profile of the patient. Given that protein aggregation is a unifying feature of ALS, we hypothesized that the underlying process of aggregate formation, and the compartment to which the aggregates are driven would be similar, even though the individual proteins involved may differ. If so, this hypothesis may provide common therapeutic strategies across all ALS variants.

We investigated three fALS-associated genes which encode proteins previously observed to accumulate in ubiquitylated inclusions in postmortem tissue. While TDP-43 positive inclusions are observed in sALS and TDP-43 fALS cases, the inclusions in SOD1 and FUS associated fALS are TDP-43 negative, and SOD1 and FUS positive respectively. Our results confirm that SOD1 is a JUNQ-like aggregate compartment substrate^30^,^31^,^33^. Expression of mutant SOD1 results in the microtubule-dependent appearance of large juxtanuclear conglomerate inclusions that always co-localize with ubiquitin (Table 1). As observed in post mortem tissue, the co-aggregation of SOD1 and TDP-43 is relatively rare in cells expressing both mutant SOD1 and TDP-43^wt^.

In contrast, TDP-43 and FUS form distinct, microtubule-independent inclusions that do not initially associate with ubiquitin in cell culture. In addition, TDP-43 and FUS inclusions are also structurally distinct from each other, as TDP-43 inclusions are immobile while FUS inclusions contain a mobile fraction (see Table 1). Neither of these inclusions fit the current definition of known aggregate compartments, including aggresomes, IPOD and JUNQ, suggesting the engagement of distinct protein quality control elements to assemble the inclusions. This may be an inherent property of the solution states of the proteins in question given we observed that the diffusion properties of non-aggregated FUS and TDP-43 were vastly different to that of SOD1. FRAP measurements of SOD1 indicate it has a rapidly diffusing element, consistent with previous work suggesting it is predominantly dimeric in cells, with mutations promoting monomer formation^33^. In contrast, FRAP measurements of non-aggregated TDP-43 and FUS (mutant and wt) indicate a very slowly diffusing species consistent with very large complexes, likely with mRNA or in the case of nuclear protein, DNA. The inherent property of TDP-43 and FUS to form functional aggregates may underlie the aggregation of these slowly diffusing species in a manner similar to stress granule formation^48^. Removal of stress granules is, at least in part, regulated by autophagy pathways^49^, and dysfunction of autophagy results in TDP-43 inclusion formation^46^. This contrasts the UPS-dependent pathways involved in misfolded SOD1 degradation^50^ and may partially explain the differences in the way SOD1, FUS and TDP-43 inclusions are formed.

While ubiquitin is thought to be universally present in TDP-43 inclusions, data from our models indicate that ubiquitin joins the inclusions late in the process. This is reminiscent of Htt inclusions^32^, and may represent ubiquitylation for degradation via autophagy rather than for degradation by the proteasome. This is consistent with the presence of autophagosomes in close proximity to the surface of inclusions in ALS post mortem tissue^51^. Interestingly, both K48 and K63 linked ubiquitin chains, directing proteins to the proteasome and autophagy pathways respectively, have been shown to localize to TDP-43 inclusions^46^ suggesting that autophagic processes may be active at these sites. While our model shows a minority of cells containing TDP-43 inclusions that co-localize with ubiquitin, most TDP-43 positive inclusions in human ALS spinal cord contain ubiquitin. However, our work shows that smaller, presumably early-stage aggregates do not overlap with ubiquitin staining in ALS post-mortem spinal motor neurons, consistent with our cell model and the concept that TDP-43 is ubiquitylated late in the aggregation process.

Specific proteins are consistently sequestered to very specific aggregation compartments in the cell. For example, proteins containing polyQ expansions have been shown to reliably deposit in IPOD-like inclusions in mammalian cells^30^,^31^,^33^. In addition, SOD1 and proteins with polyA expansions are consistently found in JUNQ-like aggregate compartments in cells^30^,^31^,^33^. Interestingly, when SOD1 is modified by fusion to a polyQ repeat it can be directed to the IPOD compartment, but without this artificial modification it consistently appears in the JUNQ compartment^31^. We confirm that while SOD1 is consistent in its cellular aggregation patterns, TDP-43 and FUS are not, displaying variable aggregation structures presumably via distinct aggregation pathways. We observed that TDP-43 could aggregate distinctly from SOD1, Htt and FUS in our cell models, and it could also co-aggregate with SOD1 and FUS. Interestingly, in these cases TDP-43 aggregates had altered diffusion properties in FUS inclusions but not in SOD1 inclusions, suggesting a co-aggregation process with mutant SOD1 but a secondary aggregation process in the case of mutant FUS. Our analysis showing a trend for a larger percentage of total FUS in these inclusions compared to that of TDP-43 supports this. The work presented here is also consistent with human post mortem tissue where TDP-43 does not accumulate with Htt in a case study of coexisting Huntington’s disease with ALS^52^ (although both polyQ repeats and TDP inclusions were present) and is not found in mutant SOD1 inclusions^27^. Similarly, FUS could co-aggregate with Htt, TDP-43 and SOD1 or it could form distinct FUS inclusions. When localized with Htt, FUS altered its diffusion properties suggesting in this case FUS aggregation was a secondary process. FUS has been found to accumulate with polyQ aggregates^53^ and with TDP-43 aggregates in human post mortem tissue^36^. This inconsistent behavior may arise due to the fact that both TDP-43 and FUS are extremely aggregation prone and may easily be pushed down several aggregation pathways within cells. Regardless, these RNA binding proteins also form inclusions that do not fit within the definitions of IPOD or JUNQ. We propose that a specific mechanism exists in cells to deal with aggregating RNA binding proteins, which we name RNA Interactor Specific Compartments/Inclusions (RISCI).

The work presented here suggests that large inclusions formed by ALS proteins are RNA independent, consistent with the formation of inclusions resulting from inappropriate misfolding related interactions rather than native interactions with RNA binding partners. The RNA binding proteins TDP-43 and FUS are intrinsically aggregation prone^38^,^54^, but while mutations in TDP-43 associated with familial ALS accelerate the rate at which it aggregates this is not the case for FUS. It is thought that the aggregation propensity of these proteins is due to the so-called prion-domain present in many RNA binding proteins^55^. In addition, mutations in SOD1 alter the protein stability and aggregation propensity in a way that correlates with disease progression in SOD1 associated fALS^56^. This intrinsic instability in these ALS proteins requires that the cell maintain protein homeostasis, either by investing in folding machinery to ensure these proteins maintain their native structure or degradation machinery to remove non-functional and misfolded protein. In general, if a cell is unable to maintain protein homeostasis it can actively partition misfolded proteins in specific ways given the individual proteins solution state, ubiquitylation state, and cellular compartment. In the current work we observe that the structures formed by TDP-43 and FUS are different, suggesting distinct pathways of inclusion formation. This is consistent with previous demonstration of fundamental differences in FUS and TDP-43 aggregation properties and a lack of overlap between genetic modifiers of FUS and TDP-43 toxicity in yeast models^38^,^57^. One possible major difference is the recent discovery that FUS forms porous hydrogels^48^ to which TDP-43 can bind; this is consistent with our work presented here that shows that FUS inclusions in cells have a diffusible component and that TDP-43 can be recruited in to these diffusible structures. While our work did not examine the relationship between aggregation and cellular dysfunction there are convincing arguments that suggest FUS and TDP-43 mutations that result in aggregates can cause either loss or gain of function^58^. Mutations in FUS can lead to inappropriate phosphorylation of RNA polymerase II^59^, loss of nuclear Gems^60^, and mutant FUS does not rescue from endogenous FUS knock down in Zebrafish^61^ consistent with toxicity from loss of function. In contrast, evidence exists to demonstrate that FUS does not need to be cleared from the nucleus to induce toxicity associated with cytoplasmic accumulation^58^ consistent with a toxic gain of function. Knock-down of zebrafish TDP-43 gene leads to a phenotype similar to that of expression of mutant human TDP-43, and can be rescued by expression of the wild type but not mutant human mutant TDP-43 consistent with at least a partial loss of function, while overexpression in yeast leads to aggregation and cellular dysfunction suggesting a toxic gain of function of aggregated TDP-43^57^. The data presented in this paper demonstrating that TDP-43, FUS and SOD1 can form structurally distinct aggregates in the cell combined with the fact that TDP-43 and FUS have very different functions to that of SOD1 but result in the same disease phenotype is consistent with a unified model of toxic gain of function ALS protein aggregates. It is tempting to speculate that one potential mechanism that could explain why distinct aggregates could be similarly detrimental to cellular function is the recruitment of other proteins (e.g. ubiquitin, optineurin, ubiquilin2) that are critical for cellular function. However, we cannot rule out that the loss of nuclear TDP-43 and FUS in to aggregates leads to a toxic loss of function. In reality the detrimental effects of mutations in these genes is likely to be a complex combination of both loss and gain of function.

Several mechanisms for forming inclusions in mammalian cells have been described, including IPOD, JUNQ^30^, aggresomes^62^, aggresome like structures (ALIS)^63^, and ER associated degradation-associated vesicles^64^. The almost ubiquitous nature of these types of inclusions in neurodegenerative disease, and the diverse and specific mechanisms that underpin their formation, reveals their importance to cellular protein quality control and human pathology. It is likely that these represent just a fraction of the pathways for aggregation in cells and further characterization of these processes is an important avenue for further investigation.

In conclusion, we observe that TDP-43 and FUS can form inclusions that do not fit the current definitions for known aggregation processes in cells and at least in some cases aggregate into RNA interactor specific compartments/inclusions (RISCI). However, they do not consistently aggregate in to one kind of inclusion type. Further understanding of the active processes promoting the formation of aggregate structures may help explain the apparent pathological heterogeneity in ALS that results in the formation of round ubiquitylated inclusion, skeins, Lewy body-like hyaline inclusions, and basophilic inclusions.

## Materials and Methods

### Plasmids

The pEGFP-N1 vectors containing human SOD1^wt^, SOD1^A4V^ and G93A SOD1 were generated as described^65^. Expression vector pCMV6-AC-GFP containing human TDP-43 and FUS was obtained from OriGene (USA). SOD1-tomato constructs were generated by replacing the GFP sequences in the GFP-tagged constructs^65^ with the tomato red fluorescent protein. TDP-43^wt^-tomato (Addgene plasmid 28205, provided by Zuoshang Xu^34^), mRFP-Ubiquitin (Addgene plasmid 11935, provided by Nico Dantuma^66^) and pmRFP-LC3 (Addgene plasmid 21075, Provided by Tamotsu Yoshimori^67^) constructs were acquired from Addgene (USA). The vectors describing Httex1(46Q) fusions (C-terminally) to mCherry and Cerulean were generated as described^33^,^68^.

### Cell Culture and Transfection

Neuroblastoma x Spinal cord hybrid NSC-34 cells^69^ were maintained in Dulbecco’s modified Eagles Medium (DMEM) supplemented with 10% foetal bovine serum (FBS, Bovogen Biologicals, Australia). Cells were kept at 37 °C in a humidified incubator with 5% atmospheric CO_2_. For confocal microscopy, cells were grown on 13 mm round coverslips in 24-well plates or on 4 or 8-well chambered coverglass (Nalge Nunc International, USA). Cells were grown in 6-well plates for cell lysate experiments.

Cells were transfected using Lipofectamine 2000 (Invitrogen, USA) according to manufacturer’s instructions with 0.5 μg DNA per well for a 24-well plate or 4-well chambered coverglass, 0.2 μg DNA per well for 8-well chambered coverglass and 2 μg DNA per well for 6-well plates. For co-transfections and triple transfections, the amount of DNA was divided equally between constructs. The DNA: Lipofectamine ratio used was 1:5 (w/v). Media was refreshed 5 h after transfection. For disruption of microtubules, cells were incubated with 33 μM nocadazole (Sigma-Aldrich, USA) overnight (~ 18 h) before analysis 48 h post-transfection.

### Cell Lysis

Transfected NSC-34 cells in 6-well plates (+/− nocadazole) were harvested 48 h post-transfection with trypsin/EDTA (Gibco, USA). Cells were washed with phosphate buffered saline (PBS) before being pelleted by centrifugation (21000 × *g* for 45 s at 4 °C). Cell pellets were resuspended in ~ 50 μl lysis buffer (600 mM KCl, 20 mM Tris-Cl (pH 7.8), 20% (v/v) glycerol, Complete^®^ Protease Inhibitor Cocktail (Roche, Switzerland)) and subjected to three freeze/thaw cycles in liquid nitrogen. To digest DNA, 250 units of DNase (Roche) was added to each lysate and incubated at 37 °C for 30 min. Lysates were then centrifuged at 300 × *g* for 5 min to pellet any large debris before the protein concentration was determined by BCA Assay.

### Filter Trap Assay

Cell lysates with a total protein concentration of 100 μg were adjusted to 200 μl with a final sodium dodecyl sulphate (SDS) concentration of 2% in PBS. Lysates were filtered through a 0.2 μm cellulose acetate membrane (Whatman, UK) pre-equilibrated in 1% SDS in PBS using a Bio-Dot SF microfiltration unit (Bio-Rad, USA) under vacuum. Large aggregates are trapped in the membrane, while soluble material passes through^70^. The membrane was blocked in heat denatured casein (HDC) for 1 h at 37 °C before probing for aggregates with either monoclonal mouse anti-GFP-HRP conjugated antibody (1:1000 in HDC, OriGene—TA150043) overnight at 4 °C or polyclonal rabbit anti-GFP (1:10 000 in HDC, Abcam, UK—ab290) overnight at 4 °C followed by anti-rabbit IgG-HRP conjugated antibody (1:2000 in HDC, Bio-Rad—170–6515) for 1 h at 37 °C.

### Laser Scanning Confocal Microscopy

Imaging of NSC-34 co-transfections was performed 24–72 hours post transfection. NSC-34 cells grown on coverslips were transfected with GFP-tagged mutant SOD1, TDP-43 and FUS and fixed for 20 min at room temperature (RT) with 4% paraformaldehyde (PFA) (Merck Millipore, USA) in PBS 48 h post transfection. Cells were permeabilised in 1% tritonX-100 (TX-100) in PBS for 30 min on ice before blocking for 30 min at RT with 5% FBS, 1% BSA 0.3% TX-100 in PBS. Cells were incubated with primary antibodies (see below) overnight at 4 °C followed by Alexa Fluor^®^-conjugated secondary antibodies reactive to mouse or rabbit IgG for 1 h on ice. All antibodies were diluted in 1% BSA, 0.1% TX-100 in PBS and cells were washed with PBS between each incubation step. Following staining, coverslips were mounted onto slides using anti-fadent mounting medium (Citifluor, UK). All cells were imaged using a Leica TCS SP5 Confocal microscope.

### Antibodies

The following primary antibodies were used for cell staining experiments: monoclonal mouse anti-ubiquitin (25 μg/ml, ab7254), monoclonal mouse anti-sequestosome 1/p62 (10 μg/ml, ab56416), monoclonal mouse anti-ubiquilin 2 (1 μg/ml, ab190283) and polyclonal rabbit anti-optineurin (12 μg/ml, ab23666). Rabbit polyclonal IgG (ab171870), purified mouse IgG2a kappa (Immunology Consultants Laboratory Inc, USA—RS-90G2A) and mouse IgG1 monoclonal antibody (Chemicon, Australia—MABC002) were used as isotype controls. All antibodies were obtained from Abcam unless otherwise specified.

### FRAP

FRAP was performed on transfected NSC-34 cells 48 h post transfection using the LAS AF FRAP Application Wizard on the Leica TCS SP5 Confocal Microscope. Images were acquired using the 63 × objective with two line averages and a scan speed of 700 Hz. Five pre-bleach images were acquired over 7.5 s with the 488 nm laser set at 20% power. The region of interest (ROI) was then bleached using the ‘zoom in ROI’ method over four frames of 1.5 s at 100% laser power. Recovery was monitored for over 1 min with the laser power set back at 20%.

### Immunostaining of post mortem tissue

Dual immunofluorescence was performed on spinal cord tissue from two SALS cases using a rabbit polyclonal anti-TDP-43 antibody (Proteintech, 10782-2-AP) and mouse monoclonal anti-ubiquitin antibody (Chemicon, MAB1510). Five μm spinal cord sections were deparaffinised with xylene, and rehydrated with a descending series of diluted ethanol and water. Antigens were retrieved by heating sections in 10 mM citrate buffer (pH 6.0). Non-specific background was blocked with 1% bovine serum albumin (BSA) (Sigma Aldrich). Sections were incubated at 4 °C overnight with the primary antibodies (1:1000 for anti-TDP-43 and 1:100 anti-ubiquitin), followed by incubation with secondary antibodies (anti-rabbit conjugated with Alexa Fluor 488 and secondary anti-mouse conjugated with Alexa Fluor 555; Life Technologies) for 1 hour at room temperature. Slides were coverslipped using the Prolong Gold antifade reagent.

Confocal fluorescence imaging was performed using a Leica DM6000 upright laser scanning confocal microscope with Leica application suite advanced fluorescence software. Images were acquired with a 40 × oil immersion objective. Images were acquired using sequential mode to avoid crosstalk between two dyes.

## Additional Information

**How to cite this article**: Farrawell, N. E. *et al.* Distinct partitioning of ALS associated TDP-43, FUS and SOD1 mutants into cellular inclusions. *Sci. Rep.* **5**, 13416; doi: 10.1038/srep13416 (2015).

## Supplementary Material

Supplementary Material

Supplementary Movie

## Figures and Tables

**Table 1:** Summary of features of the inclusions of TDP-43^M337V^, FUS^R495X^ and SOD1^A4V^.

	TDP-43	FUS	SOD1
Recruits TDP-43^WT^	✓ (88%^*^)	✓ (42%^*^)	✓ (25%^*^)
Microtubule dependent	×	×	✓
Contain a mobile fraction	×	✓	✓
Co-aggregate with Htt_ex1_46Q	×	✓	×
Co-aggregate with JUNQ substrate SOD1	✓50%^*^	✓30%^*^	✓
Co-localisation with ubiquitin	✓Late	✓Late	✓Early

*expressed as a percentage of cells containing both types of inclusions.
