# Supplementary material for: Distinct partitioning of ALS associated TDP-43, FUS and SOD1 mutants into cellular inclusions
Source: Sci Rep. 2015 Aug 21;5:13416. doi: 10.1038/srep13416 (PMC4544019; doi:10.1038/srep13416)
Supplement: Supplementary Material [file srep13416-s1.pdf]

Distinct partitioning of ALS associated TDP-43, FUS and SOD1 mutants into cellular inclusions

Natalie E. Farrawell[1,2], Isabella A. Lambert-Smith[1,2], Sadaf T. Warraich[4], Ian P. Blair[4] Darren N. Saunders[5,6], Danny M. Hatters[3], Justin J. Yerbury[1,2]^.

Supplementary Figures

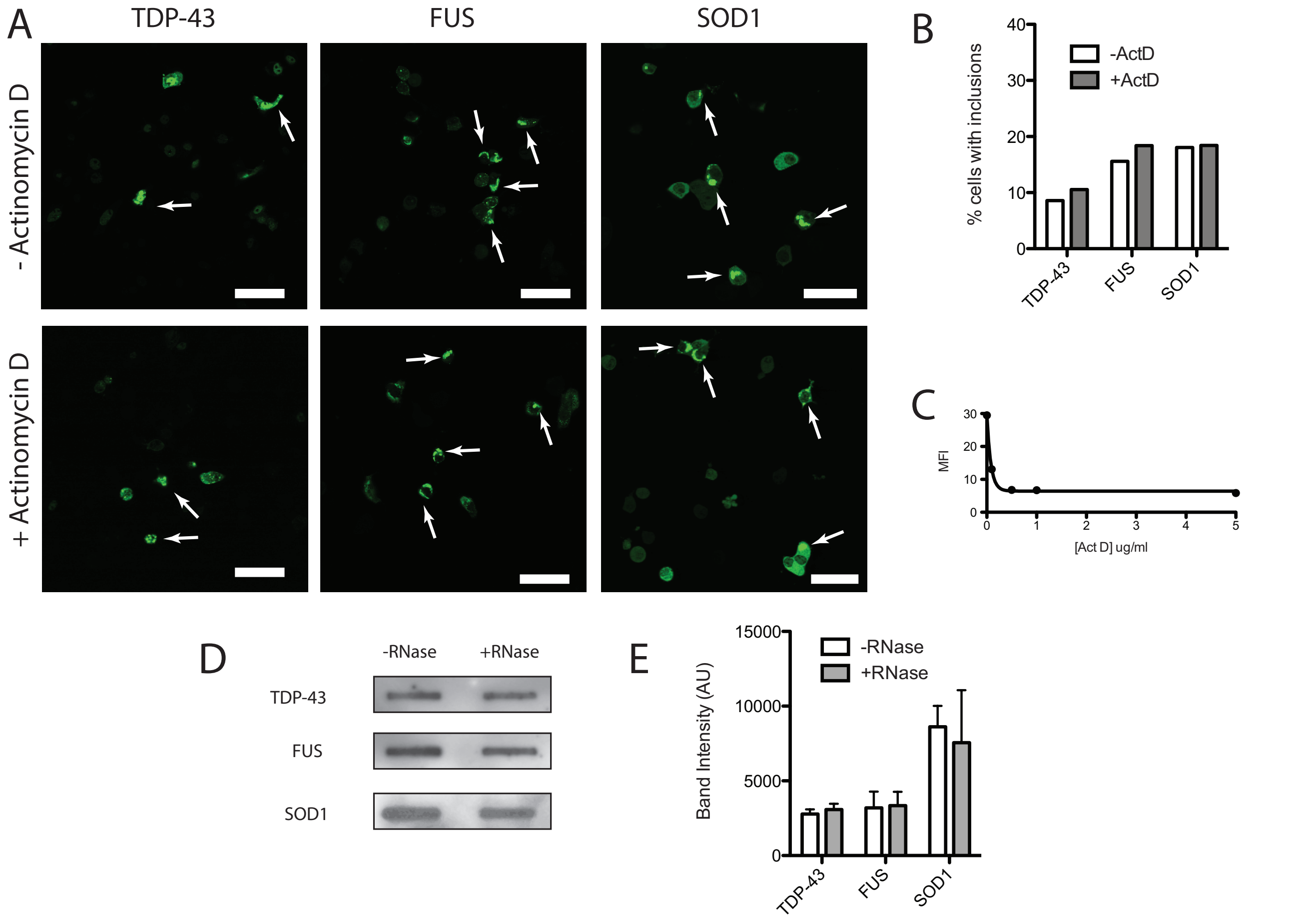

Fig. S1. Large ALS inclusions are RNA independent. (A) NSC-34 cells expressing mutant TDP-43, FUS or SOD1-GFP were treated with RNA synthesis inhibitor (5 µm actinomycin D) or not 24 h after transfection. Confocal microscopy was used to image cells 48 h after transfection. (B) Number of cells containing inclusions were counted to determine proportion of transfected cells containing large inclusions. (C) We verified that the dosage of actinomycin D completely supressed RNA synthesis during the 24 h treatment period using a Click-iT RNA synthesis kit. (D) Cell lysates from cells expressing mutant TDP-43, FUS or SOD1-GFP were treated with 2mg/mL RNase and analysed by filter trap assay. RNase treatment did not modify the size of the aggregates.

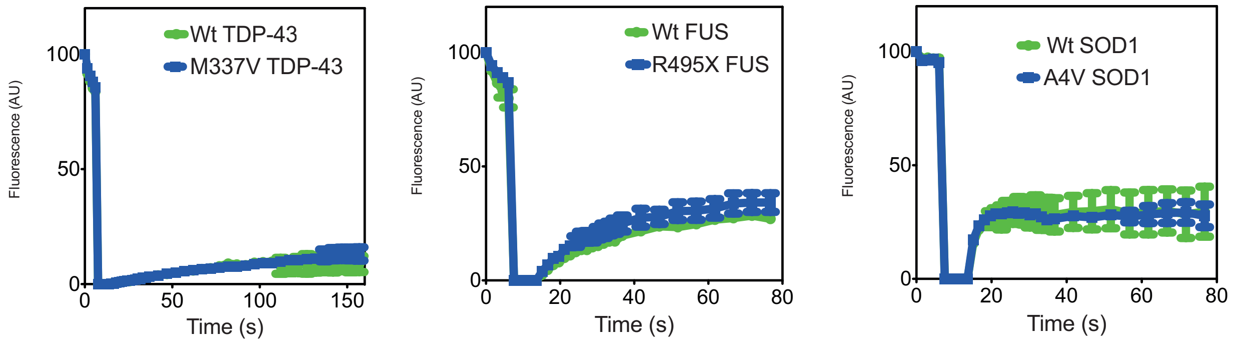

Fig. S2. FRAP analysis of soluble wt and mutant SOD1, TDP-43 and FUS-GFP tagged proteins. FRAP analysis of non-aggregated proteins in NSC-34 cells expressing mutant TDP-43, FUS or SOD1-GFP. Mean fluorescence intensity (from within the ROI) plotted over time. Prebleach intensity was recorded, and recovery was recorded for up to 150s. Results are means and standard deviation from n = 10.

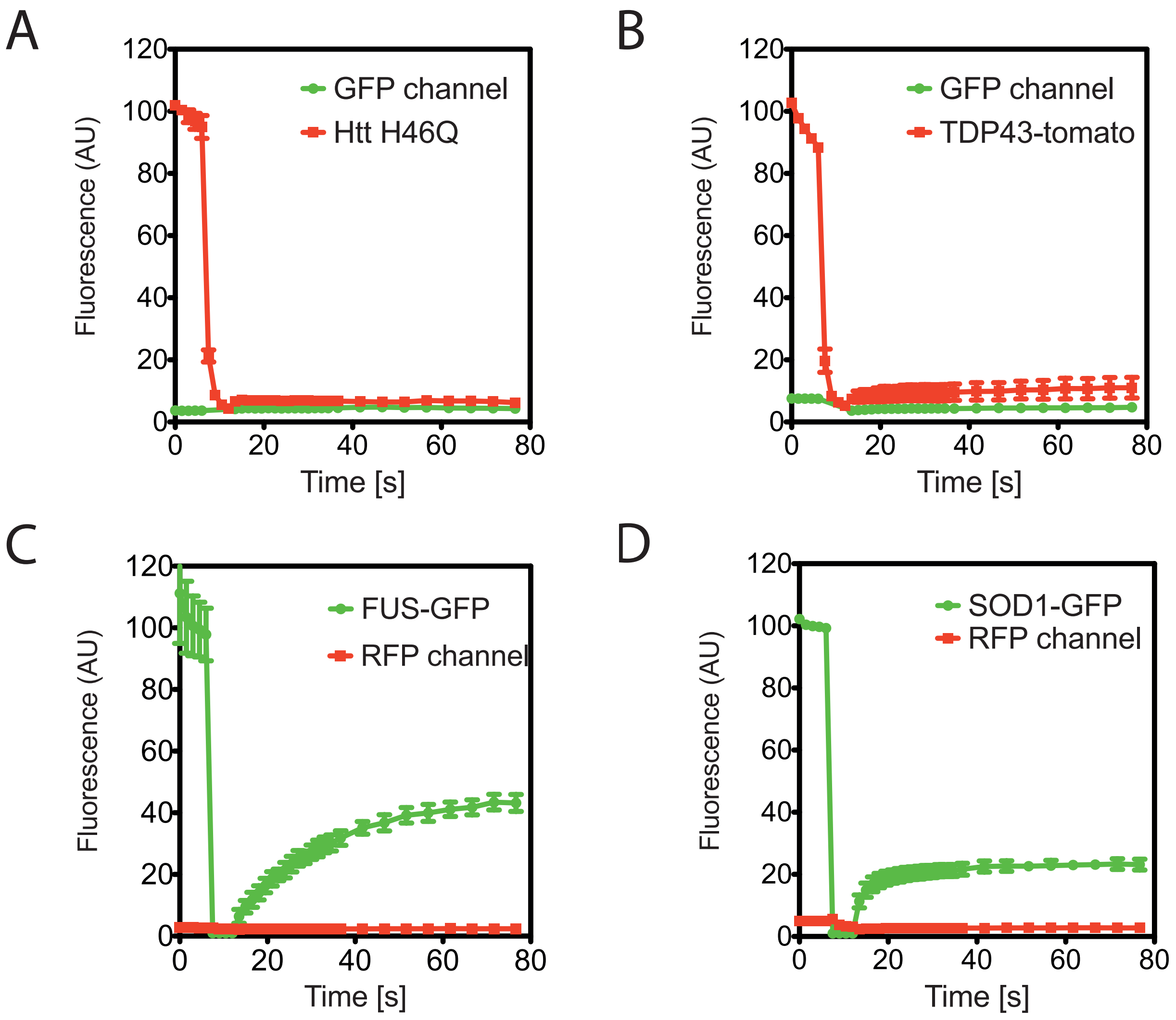

Fig. S3. FRAP analysis of inclusions containing only Htt, TDP-43, FUS and SOD1. FRAP analysis of proteins in inclusions in NSC-34 cells co-expressing Httex146Q-mcherry and either mutant TDP-43, FUS or SOD1-GFP. Mean fluorescence intensity (from within the ROI) plotted over time. Prebleach intensity was recorded, and recovery was recorded for up to 150s. Results are means and standard deviation from n = 10.

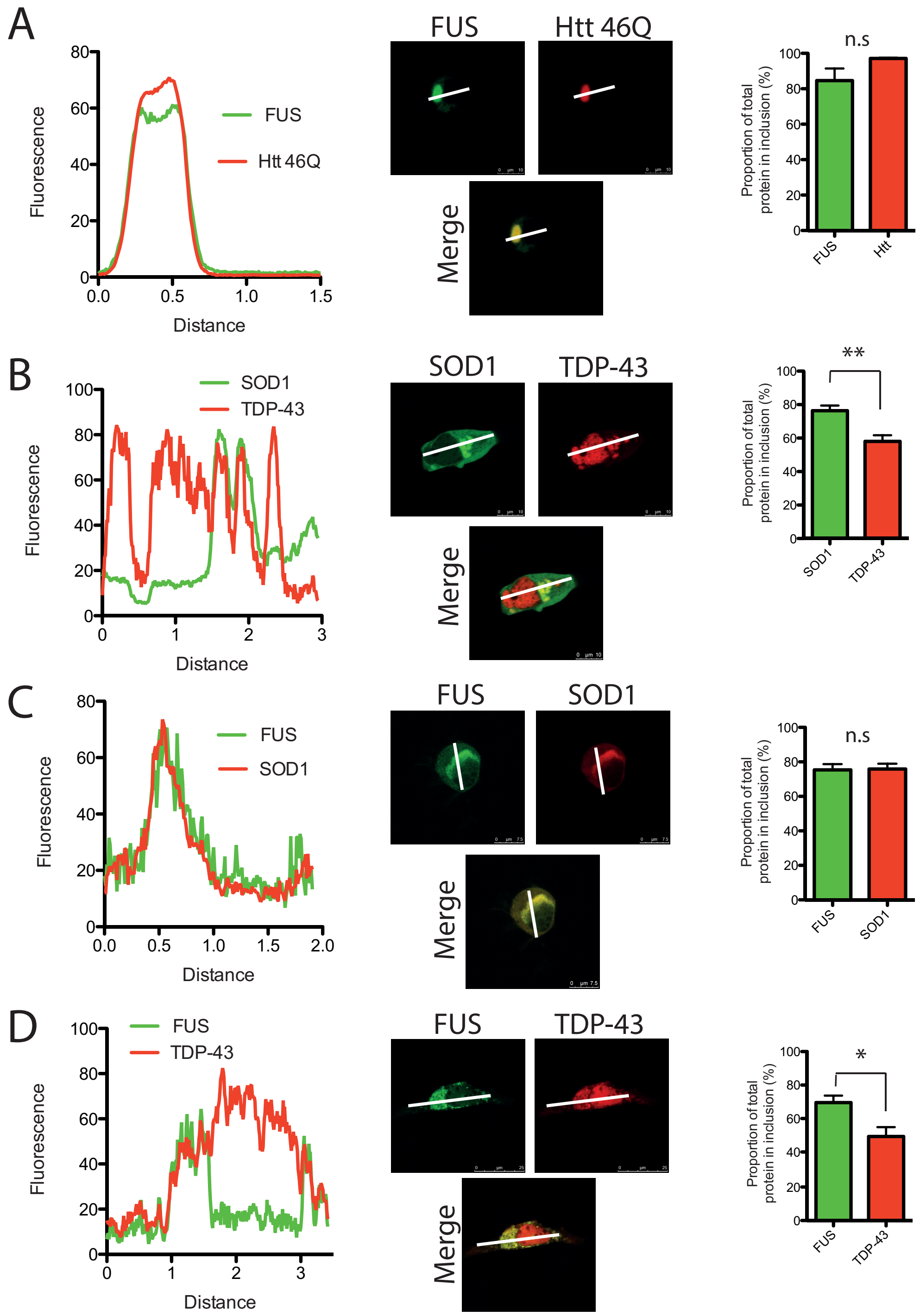

Fig. S4. Image analysis of inclusions containing combinations of Htt, TDP-43, FUS and SOD1. NSC-34 cells were contransfected with either Httex146Q-mcherry and FUS-GFP (A), SOD1-GFP and TDP-43-tomato (B), FUS-GFP and SOD1-tomato (C), or FUS-GFP and TDP-43-tomato (D). Cells containing inclusions of colocalized fusion proteins were analysed using a region of interest tool in imageJ. The proportion of total fluorescence of each fusion protein that corresponded to inclusions was estimated. Results are means and standard deviation (n=6). * indicates $p < 0.05$, ** indicates $p < 0.01$.

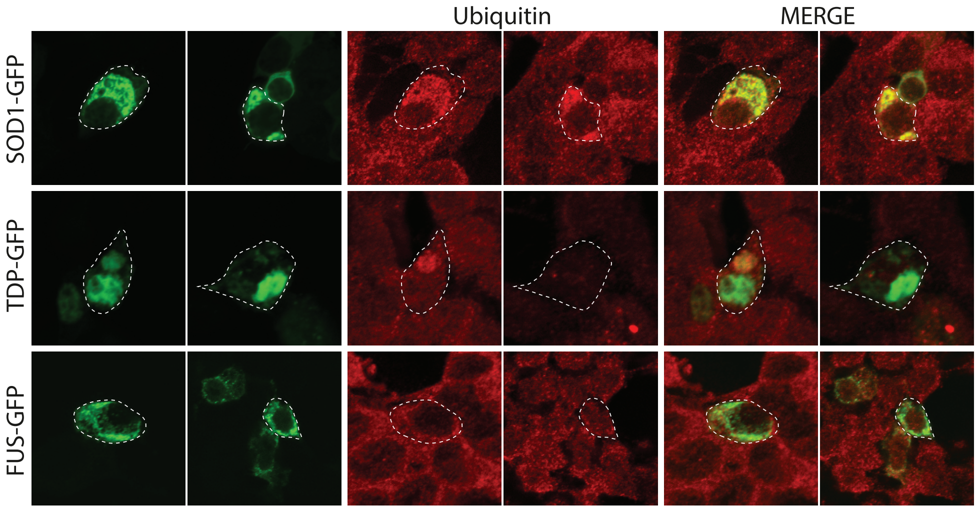

Fig. S5. Ubiquitin immunostaining confirms few TDP-43 inclusions are ubiquitin positive. NSC-34 cells were transiently transfected with mutant TDP-43, FUS and SOD1-GFP fusions and the incubated for 48-72 hours. Cells were then immunostained with antibodies raised against ubiquitin. Images shown are from 72 hours, similar results were found at 48 hours (data not shown).

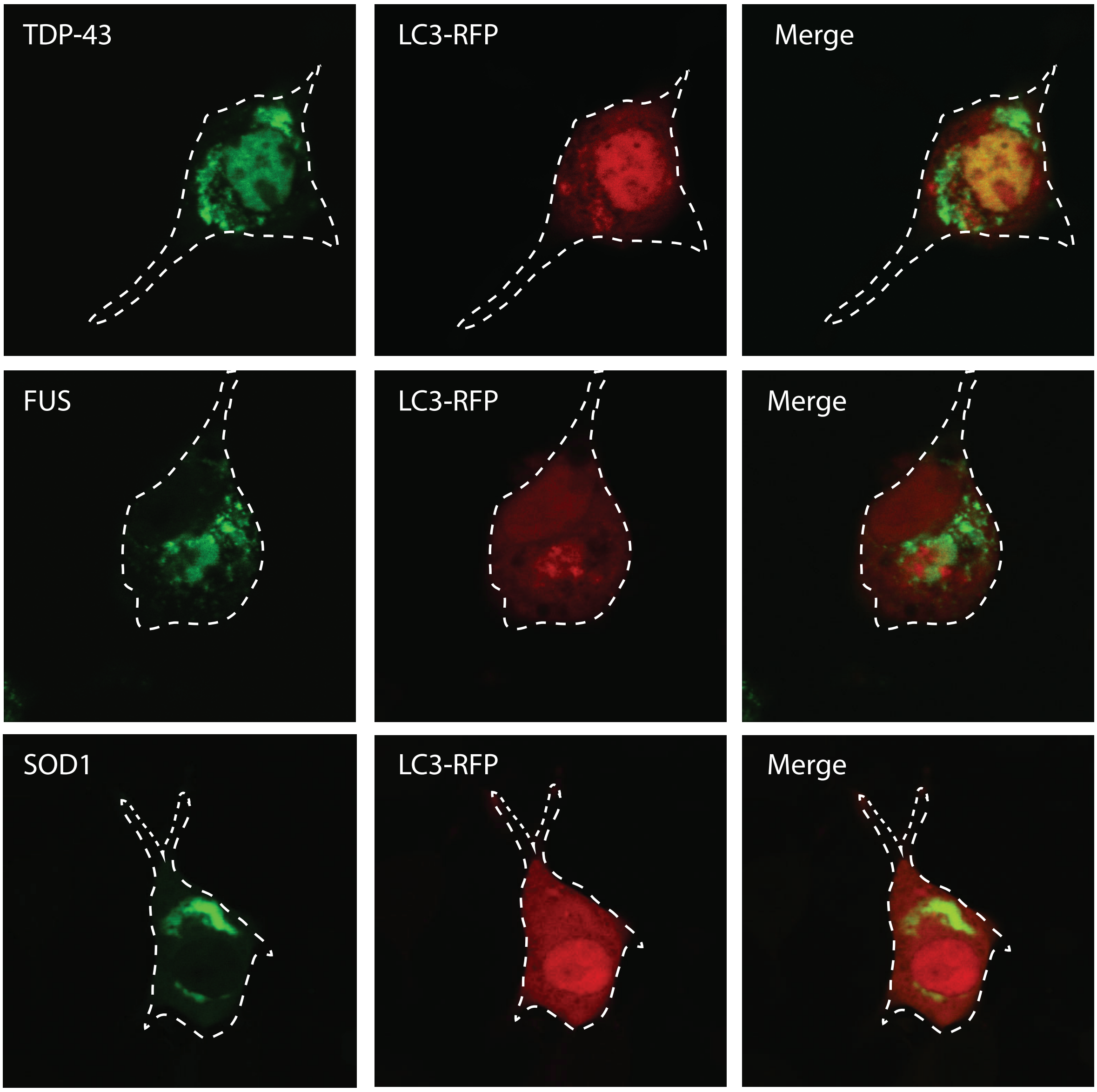

Fig. S6. TDP-43 and FUS inclusions are adjacent to LC3 foci. NSC-34 cells were transiently co-transfected with LC3-mcherry and either mutant TDP-43, FUS and SOD1-GFP fusions and then incubated for 48 hours. Cells were then imaged.

Supplementary Files

1. 3D reconstruction of Htt, TDP-43 and SOD1 Triple transfection. Z-stacks of NSC-34 cells cotransfected with SOD1-GFP, TDP-43-tomato and Htt-CFP imaged 48 h post transfection. Red dot was used as a counter stain (coloured Blue in this reconstruction). TDP-43 formed inclusions that did not overlap with GFP, CFP or red dot nuclear stain.
